# Facilitators and barriers in implementing the Minimum Initial Services Package (MISP) for reproductive health in Nepal post-earthquake

**DOI:** 10.1186/s13031-018-0170-0

**Published:** 2018-08-15

**Authors:** Anna Myers, Samira Sami, Monica Adhiambo Onyango, Hari Karki, Rosilawati Anggraini, Sandra Krause

**Affiliations:** 1 Women’s Refugee Commission, 15 W 37 Street, 9th Floor, New York, NY 10018 USA; 2 Johns Hopkins Bloomberg School of Public Health, 615 N Wolfe St, Baltimore, MD 21205 USA; 3 Boston University School of Public Health, 801 Massachusetts Avenue, 3rd floor, Boston, MA 02118 USA; 4 United Nations Population Fund, Patan, Kathmandu, 44600 Nepal; 5 United Nations Population Fund, Menara Thamrin 7th floor, JL MH Thamrin kav 3, Jakarta, Indonesia

**Keywords:** Reproductive health, Minimum initial services package (MISP), Nepal earthquake; emergency response

## Abstract

**Background:**

Following the Nepal earthquake in April 2015, UNFPA estimated that 1.4 million women of reproductive age were affected, with approximately 93,000 pregnant and 28,000 at risk of sexual violence. A set of priority reproductive health (RH) actions, the Minimum Initial Services Package (MISP), was initiated by government, international and local actors. The purpose of this study was to identify the facilitators and barriers affecting the implementation of priority RH services in two districts.

**Methods:**

In September 2015, a mixed methods study design was used in Kathmandu and Sindhupalchowk districts to assess the implementation of the priority RH services five months post-earthquake. Data collection activities included 32 focus group discussions with male and female participants aged 18–49; 26 key informant interviews with RH, gender-based violence (GBV), and human immunodeficiency virus (HIV) experts; and 17 health facility assessments.

**Results:**

The implementation of priority RH services was achieved in both districts. In Kathmandu implementation of emergency RH services started within days of the earthquake. Facilitating factors for successful implementation included disaster preparedness; leadership and commitment among national, international, and district level actors; resource mobilization; strong national level coordination; existing reproductive and child health services and community outreach programs; and supply chain management. Barriers included inadequate MISP training for RH coordinators and managers; weak communication between national and district level stakeholders; inadequate staffing; under-resourced and fewer facilities in rural areas; limited attention given to local GBV and HIV organizations; low availability of clinical management of rape services; and low awareness of GBV services and benefits of timely care.

**Conclusion:**

Ensuring RH is included in emergency preparedness and immediate response efforts and is continued through the transition to comprehensive care is critical for national governments and humanitarian response agencies. The MISP for RH remains a critical component of response efforts, and the humanitarian community should consider these learnings in future emergency response.

## Background

Reproductive health (RH) services in humanitarian emergencies remain a critical priority. As greater numbers of people become displaced and the contexts of their displacement becomes more diverse, ensuring access to the standard of care of priority RH services must be included in all types of emergency response efforts. Additionally, addressing comprehensive RH care when transitioning out of the emergency phase is vital. The priority RH services, known as Minimum Initial Services Package (MISP) for RH, were identified in 1996 and included in the Reproductive Health in Refugee Situations: An Inter-agency Field Manual (IAFM) in 1999 [1]. The MISP became part of the Sphere Minimum Standards in 2004 [2]. The MISP is to ensure the availability of RH services that have the most impact on reducing RH-related morbidity and mortality in the early days and weeks of new emergencies. The MISP is a coordinated set of activities designed to prevent and manage the consequences of sexual violence, reduce transmission of human immunodeficiency virus (HIV), prevent excess newborn and maternal morbidity and mortality, and to plan for comprehensive RH services. Additional priority activities include: access to contraception for existing users; syndromic management of sexually transmitted infections (STIs); continued access to antiretroviral (ARV) drugs for those in need, as well as prevention of mother-to-child transmission (PMTCT) of HIV; and access to menstrual hygiene supplies [3].

Through the Inter-agency Working Group (IAWG) for Reproductive Health in Crisis, assessments of the degree to which the priority RH services have been implemented in an emergency response have been conducted in Indonesia (2005), Kenya (2008), Haiti (2010), and Jordan (2013) [4, 5]. Increasingly, RH services have been included in emergency response, with higher levels of awareness of RH priorities among lead agencies and actors. However, there has been slower implementation of services in certain areas such as in harder-to-reach locations and services related to GBV [4, 5]. Assessments in Haiti revealed increased awareness of RH priorities by lead international agencies compared to previous MISP assessments in Kenya and Indonesia. Nonetheless, gaps in funding, service provision, and information dissemination led to weak GBV prevention and response efforts and inconsistent comprehensive emergency obstetric care (CEmoC) and newborn care in Haiti [4]. In Jordan, awareness of priorities was high, as was funding, but shortfalls existed in demand for services, perceived low quality of maternal and newborn care, and weak provision of GBV response services [5]. In both contexts, established pre-crisis health services allowed for stronger responses around HIV services in Haiti, and availability of maternal and newborn care in Jordan [4, 5].

### Nepal context

On April 25, 2015, an earthquake of 7.8 magnitude hit Nepal, followed by more than 300 aftershocks including one of 7.3 magnitude on May 12, 2015 [6–8]. Thirty-five of the 75 districts in the country were affected, close to 9000 people died, and over 22,000 people were injured [9, 10]. The total number of those displaced reached 2.8 million with around 600,000 houses destroyed and 290,000 damaged [9, 10]. UNFPA estimated that 5.6 million people were affected, including 1.4 million women of reproductive age [9]. At the time of the earthquake, approximately 93,000 women were estimated to be pregnant, including 1000–1500 women likely to experience complications and 28,000 women estimated to be at risk of sexual violence [9]. In Kathmandu 20.1% of the district’s population (350,676 people) and in Sindhupalchowk 99.9% of the district’s population (287,574 people) were identified as in need after the earthquake [11].

Prior to the earthquake, Nepal had made advances in enabling access to health care services for its predominantly rural population, estimated at 81% of its total population [12]. Programs and strategies including the National Reproductive Health Strategy, the National Reproductive Health Commodity Security Strategy (2007–2011), the Nepal Family Health Program, the National Female Community Health Volunteer Programme, the National Safe Motherhood Plan (2002–2017), the National Policy for Skilled Birth Attendants (SBA), and the Safe Delivery Incentive Program, had been implemented. These efforts strengthened all levels of the health care system to provide family planning, safe abortion services and maternal and child health services. They also worked to reduce transmission and manage STIs including HIV and worked to improve health services-seeking behavior for RH [13–20]. Disaster preparedness and response efforts prior to the earthquake included: establishing protocols; incorporating the MISP components in disaster preparedness and response plans; prepositioning of RH kits; engaging health facilities; and conducting training of trainers with selected government and non-governmental organization (NGO) officials on emergency response, including the MISP, primarily in Kathmandu [20–25].

Studies exploring aspects of Nepal’s emergency response have highlighted that despite these preparations, response efforts were not gender sensitive, and women faced considerable challenges in addressing their RH needs including accessing menstrual hygiene supplies [26–28]. Calls to leverage rebuilding efforts to expand universal health access and improve equity were also raised, as was the need for better coordination and planning of services to avoid duplication [29, 30].

The purpose of this study was to assess the implementation of the priority RH services (that reflect the MISP components) in Nepal in two districts: Sindhupalchowk and Kathmandu. The objectives were to: 1) describe factors that facilitated implementation; 2) explain the barriers for implementing RH services; and 3) recommend solutions for improving RH response in Nepal. The study was conducted by a team from the IAWG on RH in Crises in September 2015, with results disseminated in reports to key stakeholders and available online [31–33].

## Methods

### Design and setting

Mixed methods were employed including qualitative and quantitative designs to assess the implementation of priority RH services in two districts in Nepal. The study was conducted in Kathmandu and Sindhupalchowk from September 13–22, 2015. The districts were selected based on diversity of context (rural and urban), number of displaced persons, and number of people in need resulting from the earthquake [11]. Data collection was done using focus group discussions (FGD), key informant interviews (KII), and health facility assessments (HFA).

### Data collection

#### Focus group discussions

FGD sites were selected by the study team based on proximity to a health facility, which was defined as near (less than 1 h walk from a birthing facility) and far (more than 1 h). Community health workers recruited FGD participants who were women and men aged 18 to 49, were living in the selected sites, were affected by the earthquake, and who spoke Nepali. The FGDs were conducted separately by gender and age (18–24 and 25–49) with up to eight participants per group.

The discussion guides were adapted from the MISP assessment conducted in Jordan among Syrian refugees by IAWG members to fit the Nepali context, translated into Nepali and piloted in Kathmandu. Local Nepali researchers who had prior experience in qualitative research were hired and trained by the co-investigators to facilitate the focus groups. Facilitation and note taking were conducted in Nepali and then transcripts were translated and transcribed into English.

#### Key informant interviews

KII participants were recruited by the lead RH agencies in the response, and the participants were selected based on their work in RH, GBV and HIV. From these agencies, participants were selected using purposive sampling with maximum variation to account for diverse perspectives. Coordinators, managers, directors, and focal points from government bodies, UN agencies, international, national, or local NGOs working in Kathmandu or Sindhupalchowk were selected to participate.

The KIIs utilized three different semi-structured questionnaires (RH, GBV, HIV) to gather information on knowledge about the MISP and the Additional Priorities, RH concerns and needs, MISP response, disaster risk reduction including preparedness, and facilitating factors and barriers to MISP implementation. The RH and GBV tools were pilot tested among current or former staff working in RH and GBV in Nepal; and the HIV tool was piloted with an HIV expert prior to data collection. All interviews were conducted by a study team member in English.

#### Health facility assessments

Health facilities in the two districts were selected using the following criteria: served populations affected by the recent earthquakes, provided delivery and newborn services during the past six months, and accessible to the study team. An interview was conducted with the individual in-charge at each health facility. A semi-structured, quantitative questionnaire adapted from previous MISP assessments, was used to interview health providers and conduct direct observation of facilities. A study member conducted the interviews in English or with an interpreter in Nepali.

### Data analysis

Analysis was conducted by the study team member who oversaw or conducted data collection using a particular method within a specific district. Study team members residing in four locations selected the method for analysis in consideration of their experience, software or application availability, and whether it was deemed appropriate. For focus group discussions, English transcripts were coded and analyzed with themes defined a priori by the MISP components using NVivo 10. For KIIs, given the minimal qualitative data, interviews were coded in Microsoft Word with themes identified according to the components of the MISP. Quantitative data were entered the Census and Survey Processing System (CSPro) and analyzed using SPSS v.22 in alignment with the IAWG MISP online toolkit [34]. HFA data, given the small sample, were entered into Microsoft Excel and analyzed to generate descriptive statistics.

Validity and credibility for the qualitative data were addressed by the research team’s oversight and involvement in the data collection process ensuring the data collected represented multiple perspectives of different stakeholders on the same topics and findings were triangulated using three methods: participants were asked to validate responses; debriefings were held daily to avoid personal bias; and an audit trail documented the process using transcripts and memos. In addition, preliminary findings were shared at a validation meeting with the RH Sub Cluster in Kathmandu at the end of data collection. Lastly, a white paper, summary report and participant report were shared with lead RH agencies following the full analysis and final drafts were shared online [31–33].

### Ethical review

The study received ethical approval from the Nepal Health Research Council (NHRC). Additional reviews were done by the Family Health Division (FHD)/Department of Health Services (DoHS) Nepal, and the Nepal RH Sub-cluster. Verbal and written consent were obtained from participants before each discussion. Confidentiality was maintained throughout the study process.

## Results

### Study participants

#### FGDs

Thirty-two FGDs were conducted across all sites, with 16 in each district. A total of 249 people participated with 127 women and 122 men. In Kathmandu, 54 participants were 18–24 years old, and 59 participants were 25–49 years old. In Sindhupalchowk, 69 participants were 18–24 years old, and 67 participants were 25–49 years old.

#### KII

A total of twenty-four KIIs were conducted in Kathmandu and Sindhupalchowk with informants representing the DoHS, UN agencies and, international and national organizations. Thirteen KIIs were conducted in Kathmandu including seven interviewees working on RH, five interviewees working on GBV, and one interviewee working on HIV. Further, 11 KIIs were conducted in Sindhupalchowk with four interviewees working on RH, five interviewees working on GBV, and two interviewees working on HIV.

#### Health facilities

Assessments were conducted at seventeen facilities with eight in Kathmandu and nine in Sindhupalchowk. Facilities visited included public and private facilities, district level facilities, primary health care centers (PHCCs), and health posts. Participants included medical officers, nurses, and auxiliary nurse midwives (ANM) in charge of the health facility.

### Knowledge and awareness of RH priorities within the MISP

The majority (10 out of 12) of lead RH agencies key informants were aware and had knowledge of the MISP objectives, but participants from non-lead agencies for RH were less knowledgeable about specific MISP objectives, or priority activities. Fewer than half (five out of 12) of the RH KIs in Kathmandu and Sindhupalchowk had been trained in the MISP despite trainings conducted prior to the earthquake in Kathmandu.

### Coordination of the priority RH services

Coordination was led by DoHS FHD and UNFPA, and implementation of the priority RH services began within days of the earthquake in Kathmandu. In Sindhupalchowk, coordination was first established in the health cluster and then in separate RH sub-cluster meetings approximately two months after the earthquake. However, RH related agendas were included and discussed in the health cluster meeting before the RH sub-cluster was established. In Kathmandu, RH KIs reported overall inclusion of key stakeholders, such as the DoHS, UN agencies, local and international NGOs and adolescents in RH sub-cluster coordination meetings. Overall, informants (83%) reported sufficient funding to meet RH emergency needs, including the availability of all RH kits (0–13). FGD participants in Kathmandu were involved in coordination efforts to distribute supplies and identify those in need. In Sindhupalchowk, Village Development Committees (VDC) were reported to be involved in distribution activities. The effectiveness of the coordination was rated ‘good’ or ‘excellent’ by most Kathmandu RH KIs and ‘good’ by all the RH KIs in Sindhupalchowk. Areas for improvement included better communication between national and district levels regarding roles and responsibilities and more participation from district officers and representatives from HIV, GBV sectors as well as lesbian, gay, bisexual, transgender and intersex (LGBTI) communities, the private sector and local and international NGOs.

### Prevent and manage the consequences of sexual violence

All GBV KIs and the majority (10 out of 12) of RH KIs had heard of incidents of sexual violence in their districts. In Kathmandu, protection efforts varied by the level of health facility and hospital facilities had taken more steps to prevent GBV. Lighting provision in Kathmandu internally displaced person (IDP) camps was inadequate. In Sindhupalchowk, the temporary field hospital provided latrines that were sex-separated but made of tarpaulin during the first eight weeks. In Sindhupalchowk, only two of the eight health facilities visited had sex-separated latrines compared to five out of nine facilities visited in Kathmandu. All latrines were equipped with inside locks.

Of 17 facilities visited in both locations, only two had private rooms for sexual violence survivors. FGD participants did not report safety concerns in accessing health facilities. The majority of RH KIs reported that clinical management of rape (CMoR) was at least partially available; however, the HFA found that only three out of nine facilities in Kathmandu could provide CMoR. In Sindhupalchowk, only half of facilities could provide partial CMoR with the gap most often seen in the availability of post-exposure prophylaxis for HIV. Reasons given for the unavailability of service provision included the need for training, additional supplies and equipment, or the lack of clients.

Most RH and GBV KIs in both districts reported that although the GBV referral pathway included health, police, and legal actors, the quality of the referral system was a concern. Although there were information, education, and communication (IEC) campaigns to encourage reporting sexual violence, FGD participants were unaware of the referral pathway. Women in Sindhupalchowk said that they would report GBV cases to the police or women’s organizations. The great majority of FGD participants said sexual violence did not exist in their communities, although a few reported their communities had cases of intimate partner violence (IPV). Women living in Kathmandu’s displacement camps reported fear while collecting water or accessing the latrines, and in one FGD group conducted in Sindhupalchowk, women reported feelings of insecurity in their temporary shelters. However, FGD participants and health service providers all noted that reporting of GBV cases was low because of shame and stigma associated with sexual violence survivors. As one woman said in Sindhupalchowk, *“Women remain silent [so] that others would [not] know about it. Otherwise they will be blamed and disgraced, even if they are innocent.”* Only one hospital in Kathmandu and the district hospital in Sindhupalchowk treated cases of sexual violence. Although there was a protocol for CMoR, and a referral pathway, there were no standard operating procedures available at health facilities for referral of survivors of sexual violence.

### Reduce HIV transmission

RH and HIV KIs and HFA participants reported that safe blood transfusion was available in Kathmandu, but had not been available in Sindhupalchowk, since the temporary hospital ceased operation. Fifteen visited facilities had sufficient supplies for the implementation of standard precautions to be followed, and RH KIs reported at least partial availability of standard precautions with quality concerns in rural facilities. Only two hospitals, one in each district, provided post-occupational exposure treatment for staff. Knowledge on HIV and STI prevention and risk reduction varied among FGD participants. More awareness was found among 18–24-year-old age groups, and male participants in Kathmandu. The location of services was less known by participants, with many reporting that the stigma of persons living with HIV might prevent people from seeking treatment. As one man in Kathmandu reported, *“If they get such disease, people are scared what society will say about them. Uneducated people hide the disease, but educated people treat it.”* Nearly all RH and HIV KIs and FGD participants reported condoms were available for free, except for FGD participants in a Kathmandu displacement camp.

### Prevent excess maternal and newborn morbidity and mortality

In Kathmandu, female participants had greater knowledge of danger signs during pregnancy compared to male participants. All participants knew where to go for maternal and child health (MCH) needs following the earthquake. FGD participants in Kathmandu preferred facility deliveries, whereas preferences varied in Sindhupalchowk and these findings aligned with interviews with facility staff. All facilities visited for the HFA were providing normal delivery services, and RH KIs reported basic emergency obstetric care (BEmOC) was available. CEmOC was available in Kathmandu but not reliably in Sindhupalchowk after the closing of the temporary hospital. Among the RH KIs, one-fourth had heard of incidents of maternal mortality, and one explained this was worse post-earthquake due to landslides, floods, and road and airport obstructions. Perceptions among FGD participants in Sindhupalchowk reflected the sense of risk due to road closures. One woman in Sindhupalchowk described, “*We have problems of the road. We have to carry the patient. Sometimes in a bed sheet, sometimes on our back. We have to carry till the road where the ambulance can come.”* The majority of KIs (10 out of 12) mentioned ensuring the availability of SBAs and supplies for normal births as priority activities, and far fewer mentioned ensuring the availability of BEmOC and newborn care (5 out of 12) or CEmOC and newborn care (6 out of 12). Just three out of 12 KIs mentioned ensuring a 24-h, seven days per week referral system to support EmOC services, although one such system had been established for pregnancy complications. RH sub cluster members reported IEC radio and TV campaigns had been conducted to increase facility deliveries. In Sindhupalchowk, after the earthquake, the referral system included the temporary hospital for CEmOC. When it ceased to operate, referrals went to Dhulikhel Hospital in the neighboring district. Sindhupalchowk FGD participants reported bypassing nearby health posts or the district hospital when experiencing complications, and going directly to the neighboring district hospital or Kathmandu for quality care. On-going barriers to access delivery care in the facilities included the direct and indirect costs associated with reaching facilities, particularly in far-away districts. Noted by one Sindhupalchowk woman, *“We go to the health post for small problems like stomach pain, but if any complication occurred we have to go to the district hospital and we cannot afford it.”* In Sindhupalchowk, RH KIs reported that female community health volunteers (FCHVs) and health facilities distributed clean delivery kits (CDKs) to pregnant women. There was newborn care, although not always comprehensive, in both districts, with well-established comprehensive care in higher-level hospitals in Kathmandu. RH KIs in Sindhupalchowk reported barriers to implementing comprehensive newborn care included lack of or malfunctioning equipment.

### Plan to integrate comprehensive RH services into primary care

KIs reported that during the immediate response, staff capacity assessments and trainings took place on RH Kits, CMoR, standard precautions, family planning, and quality assurance for maternal and newborn care. At the time of the assessment, RH KIs reported a continuing need for human resource assessments and training at the national and district levels. RH KIs reported that more birthing facilities were being identified, however with concerns that too many birthing facilities could limit provider’s practice of SBA. At the time of the assessment, nearly all visited health facilities reported a reliable supply chain. However, two-thirds of the facilities reported shortages immediately after the earthquake.

### Additional priorities of the MISP

Among FGD participants, groups reported awareness of short and long-term contraceptive methods, except for emergency contraception (EC), and knowledge of free services. A shortage of injectables and implants following the earthquake was filled by UNFPA, although the availability of EC continued to vary between and within districts. The HFA showed that all but two facilities (one in each district), provided syndromic treatment for STIs; however, nearly all facilities reported an insufficient supply of needed medicines for this care, significantly affecting quality. Most FGD participants had low knowledge of STIs or location of services. Most RH and HIV KIs reported that antiretroviral (ARV) therapy had continued, without delay, and referral systems were reportedly functioning. The HFA found that all visited facilities in Sindhupalchowk had a referral system for ARV therapy, including PMTCT. In Kathmandu, four facilities reported a referral system. Although all RH KIs reported that supplies for menstrual hygiene were at least partially available if not fully available through mobile services, availability varied between districts. In Kathmandu, only the two displacement camps visited reported having received menstrual hygiene supplies, compared to all FGDs except one in Sindhupalchowk. Participants asked for more guidance on disposal and for more supplies.

There was little reporting of inclusion of LGBTI persons, adolescents, or persons with disabilities in programming. Only one facility in total was equipped with accessible toilets and wheelchair ramps.

Although there was limited adolescent programming, a high interest among FGD participants was reported. RH KIs also cited adolescent corners in camps during the response. In Sindhupalchowk, adolescents could access SRH services without parental consent in 75% of visited facilities, and in Kathmandu, in a third of visited facilities.

### Disaster risk reduction, including preparedness

RH, GBV, and HIV KIs reported a number of RH-related emergency preparedness activities prior to the earthquake, including incorporation of MISP components in disaster preparedness and response plans; the pre-positioning of RH Kits; development of a logistics system; MISP trainings and emergency drills; facility assessments; emergency preparation by hospitals; establishment of a Code of Conduct (CoC) on sexual exploitation and abuse (SEA) prevention; inclusion of GBV in national disaster policy, with pre-positioning of post-rape kits and a GBV referral system; HIV prevention trainings; and a national-level pre-positioning of condoms and medicines. However, logistical challenges were reported by RH KIs during response efforts.

## Discussion

These findings are further evidence of a global, positive trend in addressing the MISP for RH priorities from the onset of emergency response efforts, as also shown in recent MISP assessments conducted in Indonesia, Kenya, Haiti and Jordan [4, 5]. These findings further highlight facilitating factors and barriers that can be critical lessons to draw upon for disaster preparedness. Our purpose was to unpack these factors that influence implementation of the MISP to enable a deeper understanding of humanitarian operations for improved decision-making at the national and global levels.

### Facilitators

The strength of the coordination of the priority RH services was likely due to the leadership commitment and existing emergency preparedness mechanisms that had been developed prior to the earthquake. Strong committed leadership is evidenced in immediate and ongoing coordination; concerted innovative efforts to address all of the MISP priority objectives and related priority activities; successful fundraising and procurement of supplies; ensuring a lead agency within the health cluster to support coordinated efforts to address the MISP -- all of which are established priority activities for the first objective of the MISP. The National Strategy for Disaster Risk Management, developed in 2008, made strides in preparation, planning, and policies that allowed for a stronger response to the earthquake than otherwise would have been possible [21]. For RH, this included training of health service providers on MISP and preparation of emergency RH kits and supplies. MISP coordination immediately after the earthquake was effective due to action on pre-established government regulations and committed leadership from the Ministry of Health (MOH)/FHD/ Epidemiology and Disease Control Division (EDCD), and the leveraging of existing relationships and working groups between government, international non-governmental organizations (INGO), UN agencies, and NGOs including RH sub-cluster members. Prior to the earthquake, the RH sub-cluster, imbedded in the health cluster as part of the mandate of the Health and Nutrition Cluster, was well represented. Immediately after the earthquake the RH sub-cluster was formally activated, and the strong leadership enabled effective coordination of the MISP in Kathmandu during the earthquake response. In Sindhupalchowk, the District Health Office led the response efforts; including establishing temporary hospitals, mobile RH camps, and distribution mechanisms, allowing the MISP to be integrated in efforts to reach more rural and remote communities. In addition, there was a pre-established Memorandum of Understanding between EDCD/MoH, Nepal Red Cross Society and UNFPA (the lead UN agency for RH), enabling a faster transition into emergency response. Moreover, MCH services and referral systems, coupled with efforts by FCHVs linking women to information and delivery services, allowed for stronger access to services, even in more rural areas, after the earthquake. Lastly, GBV programs and the strong leadership by the Ministry of Women, Children, and Social Welfare (MoWCSW)/Department of Women and Children in partnership with UNFPA enabled rapid coordination of protection and GBV clusters, establishment of a GBV referral pathway, and IEC campaigns. The already existing National Health Education Information and Communication Center (NHIECC) of Nepal also bolstered the campaigns.

### Barriers

Despite dedicated leadership and pre-earthquake RH coordination mechanisms, there were significant barriers to more effective implementation of priority RH services. Although the need for the priority RH activities in emergencies was understood by stakeholders, concern of the prioritization of community needs – such as food and water – became a barrier to the provision of RH services. Inclusion of GBV and HIV actors, as well as intersectoral coordination with RH efforts, was reportedly weak, as was communication between district and national level authorities including the designation of roles and responsibilities between stakeholders. There was a lack of an opportunity to implement district contingency planning before the earthquake and a gap in understanding of the contents of RH kits to implement recommended RH services. International actors’ knowledge of existing national programs and of the human resources and capacity of local organizations was limited, leading to parallel activities by international agencies. Delays in establishing an RH sub-cluster in Sindhupalchowk after the earthquake led to delays in the MISP implementation. Funding was not always available during emergency response efforts despite overall reports of adequate funding. Insufficient human resources, including too few qualified staff to offer CMoR and HIV services, led to low capacity for adequate response and quality concerns in the referral pathway. Barriers to accessibility to all RH services included low awareness of services in some communities, direct and indirect financial costs accessing services, challenging road conditions, destruction of district hospital in Sindhupalchowk, and low reporting by community members because of their own survival priorities, as well as shame and stigma associated with GBV and HIV. Logistical challenges from the earthquake and subsequent monsoon, as well as hesitation from communities to engage in outside efforts to rebuild at the compromise of their own community’s rebuilding initiatives, were also reported as barriers.

These findings contribute to the small body of peer-reviewed and grey literature exploring implementation of RH services in post-earthquake Nepal. Results vary across studies; a report by the Central Department of Population Studies found an overwhelming majority of respondents satisfied with health services, and 80% reported access to RH and women’s health information [35]. Similarly, positive, and in contrast to our results, a WHO review of Nepal’s Adolescent Sexual Reproductive Health (ASRH) Programme of Nepal and a report by UNFPA, found integration of ASRH into post-earthquake RH response and inclusion of adolescents in response efforts including RH [36, 37]. A Nepal case study, finding a largely effective response, detailed implementation of the national RH response and reported barriers from geographical access challenges, gaps in availability of RH kits, and lack of contextualized guidelines [38]. Chaudhary et al. also noted facilitators including strong commitment of RH stakeholders and national leadership allowing for quick activation of RH services, in addition to media outreach and preparedness of UN and external partners [38]. Other studies found RH response efforts to be less than adequate and identified difficulties in accessing RH services, especially menstrual hygiene supplies [26–28, 39].

### Recommendations

Multiple efforts have taken place since the earthquake to continue strengthening RH services while transitioning out of emergency response to comprehensive RH services. This transition will continue to be aided by the policies, programs, and protocols established pre-earthquake. Attention to strengthening the implementation of these mechanisms and protocols will enable improved access to more long-term FP methods; stronger quality of and awareness and access to GBV, HIV, and STI services; reduce stigma of GBV and HIV; and improved access to culturally appropriate menstrual hygiene supplies. Notably, since the study, the National Family Planning Costed Implementation Plan (2015–2020) has been established to outline Nepal’s strategy of enabling provision of and access to quality family planning services, and central and district level MISP trainings were held with efforts to mainstream MISP into district disaster preparedness and response plans [25, 40]. In addition, regarding GBV specifically, the National Protection Cluster Strategic Action Plan developed includes GBV prevention and response.

Nepal’s geographical terrain and rural communities also demand on-going attention to increasing access for harder-to-reach communities and ensuring effective staffing, referral pathways, and supply chains to enable required services. Encouragingly, since the study, more than 300 health service providers have been trained on CMoR, and three new districts, including Sindhupalchowk, now have One Stop Crisis Management Centers (OSCMCs). Lastly, commitment to more inclusive programming is needed to accommodate the unique needs of key populations such as persons with disabilities, adolescents, and LGBTI individuals.

### Limitations

The study took place approximately five months after the earthquake; therefore, some KIs involved in response had left Nepal, and the long recall period for participants might have made it difficult to accurately recall immediate, post-earthquake service availability. Additionally, FGD participants were selected based on their proximity to functioning birthing centers (not by their level of impact from the earthquake), which may have led to responses from those with greater access to services. In addition, we were unable to reach all operational health facilities in the districts due to time constraints and challenging road conditions. Lastly, this study took place in two districts and findings only reflect the RH response in these locations.

## Conclusion

Findings from Nepal contribute to a continuing global, positive trend in attention to RH priorities from the onset of an emergency. This attention was demonstrated by disaster preparedness by the government, commitment to RH before and immediately after the earthquake, a pre-existing health system and outreach program, and effectiveness of coordination particularly on a national level. The implementation of the MISP in post-earthquake Nepal provides valuable lessons for national governments and humanitarian practitioners on factors that facilitate and hinder effective implementation of critical RH services during an emergency. Considering these findings when preparing for, and responding to, crises will allow for stronger and more effective response efforts, and ultimately reducing women and girls’ excess morbidity and mortality.

## Abbreviations

ANM: Auxiliary nurse midwives
ARV: Antiretroviral
ASRH: Adolescent Sexual Reproductive Health
BEmOC: Basic Emergency Obstetric Care
CDK: Clean delivery kit
CEmOC: Comprehensive Emergency Obstetric Care
CMoR: Clinical Management of Rape
CoC: Code of conduct
CSPro: Census and Survey Processing System
DoHS: Department of Health Services
EDCD: Epidemiology and Disease Control Division
EmOC: Emergency Obstetric Care
FCHV: Female Community Health Volunteers
FGD: Focus group discussion
FHD: Family Health Division
FPAN: Family Planning Association of Nepal
GBV: Gender Based Violence
HFA: Health Facility Assessment
HIV: Human Immunodeficiency Virus
IAWG: Inter-agency Working Group on Reproductive Health in Crises
IDP: internally displaced person
IEC: Information, Education and Communication
INGO: International Non-Governmental Organization
IPV: Intimate Partner Violence
KI: Key informant
KII: Key informant interview
LGBTI: Lesbian, Gay, Bisexual, Transgender, Intersex
MCH: Maternal and child health
MISP: Minimum initial services package
MOH: Ministry of Health
MoWCSW: Ministry of Women, Children and Social Welfare
NGO: Non-governmental Organization
NHIECC: National Health Education Information and Communication Center of Nepal
OSCMC: One-Stop Crisis Management Center
PHCC: Primary Health Care Center
PMTCT: Prevention of Mother to Child Transmission
RH: Reproductive Health
SEA: Sexual exploitation and abuse
SPRINT: Sexual and Reproductive Health Programme in Crisis and Post Crisis Situations
STI: Sexually transmitted infection
UNFPA: United Nations Population Fund
VDC: Village Development Committee
